# The Effect of Work-Family Conflict on Occupational Well-Being Among Primary and Secondary School Teachers: The Mediating Role of Psychological Capital

**DOI:** 10.3389/fpubh.2021.745118

**Published:** 2021-10-28

**Authors:** Mengmeng Zhou, Dawei Wang, Lianyong Zhou, Yiying Liu, Yixin Hu

**Affiliations:** ^1^School of Psychology, Shandong Normal University, Jinan, China; ^2^Department of International Exchange and Cooperation, Shandong Normal University, Jinan, China

**Keywords:** primary and secondary school teachers, work-family conflict, occupational well-being, psychological capital, job demands-resources model

## Abstract

In this study, 223 primary and secondary school teachers in Shandong province were selected to examine the effect of work-family conflict on occupational well-being, using the questionnaire of work-family conflict, occupational well-being and psychological capital as measuring instruments. We further explored the mediating role of psychological capital between work-family conflict and occupational well-being. The obtained data were analyzed using SPSS20.0, AMOS16.0 and M-plus 7.0. Results revealed that (1) Work-family conflict was negatively correlated with the occupational well-being and psychological capital of primary and secondary school teachers, and negatively predicted occupational well-being and psychological capital of primary and secondary school teachers; (2) Psychological capital had a significant positive correlation with the occupational well-being of primary and secondary school teachers, and significantly predicted the occupational well-being of primary and secondary school teachers; (3) Psychological capital of primary and secondary school teachers played a mediating role in work-family conflict and occupational well-being.

## Introduction

As early as 1985, Greenhaus and Beutell (1) made a clear definition of work-family conflict. They pointed that work-family conflict was a kind of role conflict, in which the role of individuals played in the work (family) made it impossible to play effectively in the family (work), giving rise to work-family conflict (1). In recent years, work-family conflict has attracted more and more attention of researchers at home and abroad (2–6). As a special stressor, work-family conflict had a negative impact on individual work and life, which was reflected not only in employees, but also in the group of primary and secondary school teachers (7). The nature of primary and secondary school teachers' work has changed. In the past, the teaching mode of schools was traditional single teaching mode. With the development of the times, primary and secondary education paid more attention to “*suzhi jiaoyu*” (8), rather than a single traditional teaching (9). This was also a challenge for primary and secondary school teachers. At the same time of working, family responsibilities should also be taken into account, resulting in the inability to achieve an effective balance between family and work, causing work-family conflict among primary and secondary school teachers. It can be seen that work-family conflict had an important impact on the work and life of primary and secondary school teachers. Well-being, as the chasing of individual, is the goal in individual living. How did work-family conflict affect the occupational well-being of primary and secondary school teachers? What is the mechanism of work-family conflict affect on primary and secondary school teachers' occupational well-being? With the introduction of positive psychology and positive organizational behavior, psychological capital, as a positive psychological factor, might be the mechanism on which work-family conflict affected the occupational well-being of primary and secondary school teachers. Therefore, the present study explored the impact of work-family conflict on occupational well-being among primary and secondary school teachers, further explored the mediating role of psychological capital in this relationship.

## Literature Review and Hypothesis

### The Relationship Between Work-Family Conflict and Occupational Well-Being

At present, the research on the relationship between work-family conflict and well-being mainly focused on three groups: employees, medical staff, and teachers (10–14). Researchers used longitudinal data to analyze and found that work-family conflict had a negative impact on employees' well-being (15). However, Neto et al. (15) only studied the general well-being of employees, while the research on professional women which conducted by Aazami et al. (16), dividing well-being into three factors: psychological depression, work satisfaction, and family satisfaction, showed that work-family conflict was related to the three sub-factors of well-being. With primary and secondary school teachers as a special kind of staff, and women accounting for the majority of this group (17), the impact of work-family conflict on their occupational well-being also attracted the attention of researchers. Previous studies on the relationship between work-family related factors (such as work-family conflict, work-family policies, well-being) among employees and physicians, found that work-family related factor positive related with turnover intention, negative related with job satisfaction and well-being (11, 18–20). The results of a study on primary and secondary school teachers also showed that individuals with higher work-family conflict felt lower job satisfaction (21).

Previous researches on the impact of primary and secondary school teachers' work-family conflict on well-being could be summarized into two aspects: first, work-family conflict impacted job-related well-being among primary and secondary school teachers. Existing studies found that primary and secondary school teachers' work-family conflict was negatively correlated with their job engagement (22–24), job satisfaction (Almutair, 2017) and was positively correlated with turnover intention (25) and job burnout (26). While job-related well-being including job satisfaction, job engagement, and job burnout, which indicated that there was a significant correlation between work-family conflict and job well-being of primary and secondary school teachers (27). Moreover, previous studies found that employees with high well-being had higher performance, lower turnover rate (28) and higher job engagement (29). In addition, there was a negative correlation between employees' well-being and job burnout (30). It can be seen from the above research that work-family conflict was related to the factors of job well-being.

Second, work-family conflict affected life well-being among primary and secondary school teachers. Researchers found that work-family conflict negatively affected the subjective well-being of professional women (31), while studies showed that the sex ratio of primary and secondary school teachers tended to be more feminized (17). Other researchers also found the negative effects of work-family conflicts on family satisfaction (32) and mental health (33). Previous researches on well-being mainly focused on job well-being, however, the well-being of primary and secondary school teachers should include not only job well-being, but also the other aspects, for example, life well-being. According to the definition developed by Zheng et al. (34), occupational well-being is divided into three basic aspects: life, work, and psychological well-being in both work and life aspects. We propose that occupational well-being involves not only employees' (including teachers) perceptions and feelings about their work and life satisfaction but also their psychological experience and the level of satisfaction exhibited in both their work and personal lives. Therefore, the occupational well-being concept consists of three dimensions and encompasses life well-being, work well-being, and psychological well-being in the present study. What was the relationship between work-family conflict and occupational well-being in the current study?

To sum up, previous studies on work-family conflict and well-being mainly focused on job, psychological, life, and the general well-being, with more emphasis on job well-being, especially. The present study including psychological well-being, job well-being, and life well-being in occupational well-being explored the relationship between work-family conflict and occupational well-being.

*Hypothesis 1: primary and secondary school teachers' work-family conflict negatively affected occupational well-being*.

### The Mediating Role of Psychological Capital

According to the Job Demands-Resources model (JDR model), proposed by Demerouti et al. (35), the characteristic of all jobs could be divided into two types. One was job-demands, and the other was job-resources. The former referred to the physical, psychological, social, or organizational demands at work, such as excessive workload, role load, role conflicts and time pressure (35). The latter referred to the physical, psychological, social, or organizational resources at work, such as social support, job feedback, rewards, and job safety (36, 37). With the consumption of job resources, there would be negative consequences (38). Primary and secondary school teachers had a heavy workload, long working hours, and numerous job demands. Work-family conflict, as a role conflict, would consume individual's physical and psychological resources, causing negative consequences such as job burnout (39). Psychological capital was put forward by Luthans et al. (40) on the basis of human capital and social capital. They pointed out that psychological capital as a positive psychological state held by an individual, which included four sub-factors: self-efficacy, hope, optimism, and resilience (41, 42). These four sub-factors promote each other and contribute to the success of individuals and organization as a whole (41). With the introduction of the concept of psychological capital, researchers at home, and abroad started to focus on psychological capital (42–44). Psychological capital, as a positive psychological factor, was a kind of psychological resources for individual (42). Therefore, primary and secondary school teachers with high work-family conflict might have a decline in the energy of psychological capital.

Researchers also found that psychological capital could improve individual well-being (45, 46). Datu and Valdez (47) conducted a study on the relationship between psychological capital and life satisfaction which belonging to life well-being and found the positive effect of psychological capital on life satisfaction. Zhao and You (45) conducted research on psychological capital and vocational well-being, indicated that psychological capital positively influenced vocational well-being through emotional labor. In addition, research on primary and secondary school teachers showed that there was a significant positive relationship between psychological capital and subjective well-being (48). Meanwhile, according to the Job Demands-Resources (JDR) model (35), excessive job demands would consume individual job resources. With the consumption of job resources, there would be negative consequences (38). Excessive work-family conflict might consume the psychological capital energy of primary and middle school teachers and lead to the decline of psychological capital. The decline of psychological capital level further led to the decline of life well-being, job well-being and psychological well-being among primary and secondary school teachers. To sum up, existing researches on the relationship between psychological capital and occupational well-being found that psychological capital was a positive predictor of occupational well-being. From the definition of Greenhaus and Beutell (1), work-family conflict contained two dimensions: work interfering with family and family interfering with work. These two dimensions would impact on the occupational well-being of primary and secondary school teachers, respectively (49).

*Hypothesis 2: Psychological capital played a mediating role in the relationship between work-family conflict and occupational well-being of primary and secondary school teachers*.

### The Present Study

**Figure 1:**
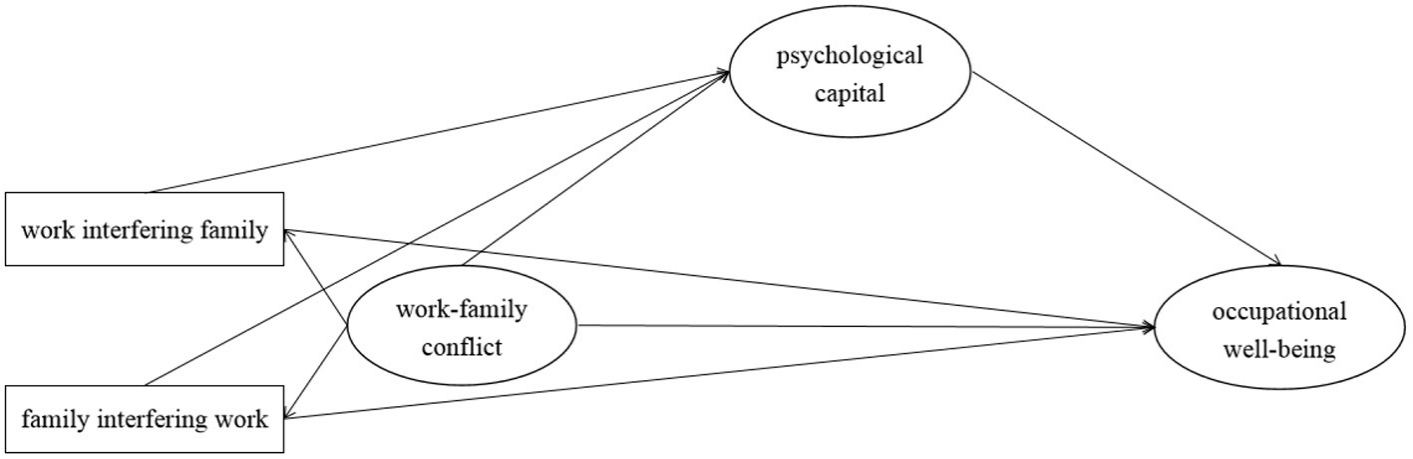
Hypothetical model of the present study.

Based on the above discussion, we speculated that work-family conflict was an important factor for psychological capital and occupational well-being of primary and secondary school teachers. High work-family conflict was an important factor for the decrease of psychological capital and occupational well-being of primary and secondary school teachers, and the decrease of psychological capital energy further reduced the occupational well-being of primary and secondary school teachers. Therefore, the present study intended to establish a mediating model, as shown in Figure 1, to explore the role of psychological capital played in the relationship between work-family conflict and occupational well-being of primary and secondary school teachers.

## Materials and Methods

### Participants and Procedure

The present study adopted cluster random sampling, taking primary and secondary school teachers from four primary and secondary schools in Shandong Province as the participants, carried on the questionnaire survey. For this study, we used a paper questionnaire to test. The teachers of each school were concentrated in one room. First, the experimenter reads out the instructions, and then two assistants distribute the questionnaire. After the teachers fill in the questionnaire, the assistants check that there are no missing questions before teachers leave their seat.

In this study, all procedures involving human participants met with the ethical standards of Academic Board of Shandong Normal University, as well as the 1964 Declaration of Helsinki and subsequent amendments. Before proceeding, participants signed the informed consent and were informed that they could withdraw participation at any time. In addition, participants were told that their participation was voluntary and anonymous.

A total of 235 questionnaires were sent out and 223 were effectively received, with an effective rate of 93.6%. Among them, 75.3% were female and 24.7% were male. With regard to age, 18.9% were under 25 years old, 44.9% were 26–30 years old, 25.1% were 31–35 years old, 7.6% were 36–40 years old, 3.1% were 41–50 years old, and 0.4% were more than 50 years old. Regarding working years, 18.4% of the participants had worked for <1 year, 23.3% for 1–3 years, 22.9% for 4–6 years, 15.7% for 7–9 years, and 19.7% for more than 10 years. 34.1% held the position of head teacher and 19.7% held the position of head of grade. With regard to marriage and childbirth, 72.2% were married and 66.4% of the participants had children.

### Measures

#### Work-Family Conflict

The work-family conflict questionnaire developed by Wu et al. (50) was divided into “work interfering with family” (WIF) and “family interfering with work” (FIW). The 22 questions rated on a 5-point scale, with 1 representing “rarely” and 5 representing “always.” Such as “I will inadvertently treat my family in the way of students, but my family doesn't accept it.” The higher the score, the greater the conflict level. The coefficient alpha for the present study was 0.911. The coefficient alpha of WIF and FIW sub-scale were 0.870 and 0.859, respectively.

#### Occupational Well-Being

The 18-item questionnaire developed by Zheng et al. (34) was adopted to measure employees' occupational well-being. Such as “I am in a good life situation,” “I find real enjoyment in my work” and “I generally feel good about myself, and I'm confident.” It mainly included three dimensions, namely life well-being, work well-being and psychological well-being. The scale used a 7-point scale from 1 representing “strongly disagree” to 7 representing “strongly agree.” A higher score indicated a higher level of occupational well-being. The coefficient alpha for this study was 0.945. The coefficient alpha of life, work, and psychological well-being were 0.890, 0.948, and 0.862, respectively.

#### Psychological Capital

The Psychological Capital Questionnaire (PCQ) was developed by Luthans et al. (41) and translated by Li et al. (51). This questionnaire consists of 24 items, such as “In my current job, I feel I can handle many things at the same time.” The items were divided into four dimensions, namely self-efficacy, hope, optimism, and resilience. The questionnaire was rated on 6-point scale, 1 represents “strongly disagree” and 6 represents “strongly agree.” A higher score indicated a higher level of psychological capital. The coefficient alpha for the current study was 0.943. The coefficient alpha of self-efficacy, hope, optimism, and resilience were 0.854, 0.854, 0.863, 0.857, respectively.

### Analysis

The data collected in this study were processed and analyzed using SPSS20.0, Amos 16.0 and M-plus 7.0. We use SPSS to test common method bias, descriptive statistics, correlation analysis, reliability analysis. Confirmatory factor analysis and mediation analysis were performed by M-plus and Amos.

## Results

### Common Method Bias

Common method bias is an artificial covariation between a predictor and a reference variable due to the homogeneity of the data source or rater, the measurement environment, the topic description, and the characteristics of the topic itself, which is a false correlation (52). The test of common method bias usually adopts Harman single factor technique (48, 53). This was done by performing an exploratory factor analysis of all the items on the work-family conflict, occupational well-being, and psychological capital questionnaires together to see the pre-rotation results. If the interpretation rate for only one factor or the first factor was more than 40%, then there was a serious common method bias. The current study adopted Harman single factor technique to estimate the influence of common method bias, which was the general way to examine the common method bias (42). In this study, the results found that thirteen factors were emerged, and the interpretation rate for the first factor was 27.46%, indicating that the common method bias in this study was not serious (52, 54).

### Confirmatory Factor Analysis

We investigated the measurement models for several different factors and compared them with the three-factor model. As shown in Table 1, the three-factor model was more suitable for data fitting in this study than other models, indicating that the participants could clearly distinguish among different factors.

**Table 1:** The results of confirmatory factor analysis.

**Model**	**χ^2^**	**df**	****χ^2^**/df**	**RMSEA**	**CFI**	**TLI**	**SRMR**
Three-factor model (X, Y, M)	50.531	22	2.30	0.076	0.972	0.954	0.040
Two-factor model (X+M, Y)	186.238	26	7.163	0.166	0.842	0.782	0.094
One-factor model (X+Y+M)	277.715	27	10.29	0.204	0.753	0.671	0.098

*X = work family conflict, Y = occupational well-being, M = psychological capital*.

### Correlation Analysis

As shown in Table 2, there was a significant negative correlation between occupational well-being and work-family conflict, work interfering with family and family interfering with work (*r* = −0.301, *p* < 0.01; *r* = −0.230, *p* < 0.01; *r* = −0.322, *p* < 0.01) and was a significant positive correlation between occupational well-being and psychological capital (*r* = 0.626, *p* < 0.01). Psychological capital was negatively correlated with work-family conflict, work interfering with family and work interfering with work (*r* = −0.212, *p* < 0.01; *r* = −0.203, *p* < 0.01; *r* = −0.184, *p* < 0.01). The results provided necessary prerequisites for further testing the mediating role of psychological capital in work-family conflict on occupational well-being. In addition, a paired sample *t*-test was conducted for work interfering with family and family interfering with work, the results showed that the mean value of work interfering with family was significantly higher than that of family interfering with work (*t* = 19.56, *p* < 0.001).

**Table 2:** Descriptive statistics and correlations among study variables (*N* = 223).

**Variables**	**M**	**SD**	**1**	**1(1)**	**1(2)**	**2**
1. Work-family conflict	1.99	0.58				
1(1) work interfering with family	2.38	0.76	0.918^**^			
1(2) family interfering with work	1.57	0.56	0.851^**^	0.598^**^		
2. Occupational well-being	5.28	0.92	−0.301^**^	−0.230^**^	−0.322^**^	
3. Psychological capital	4.72	0.61	−0.212^**^	−0.203^**^	−0.184^**^	0.626^**^

** *p < 0.01*.

### Hypothesis Testing

**Figure 2:**
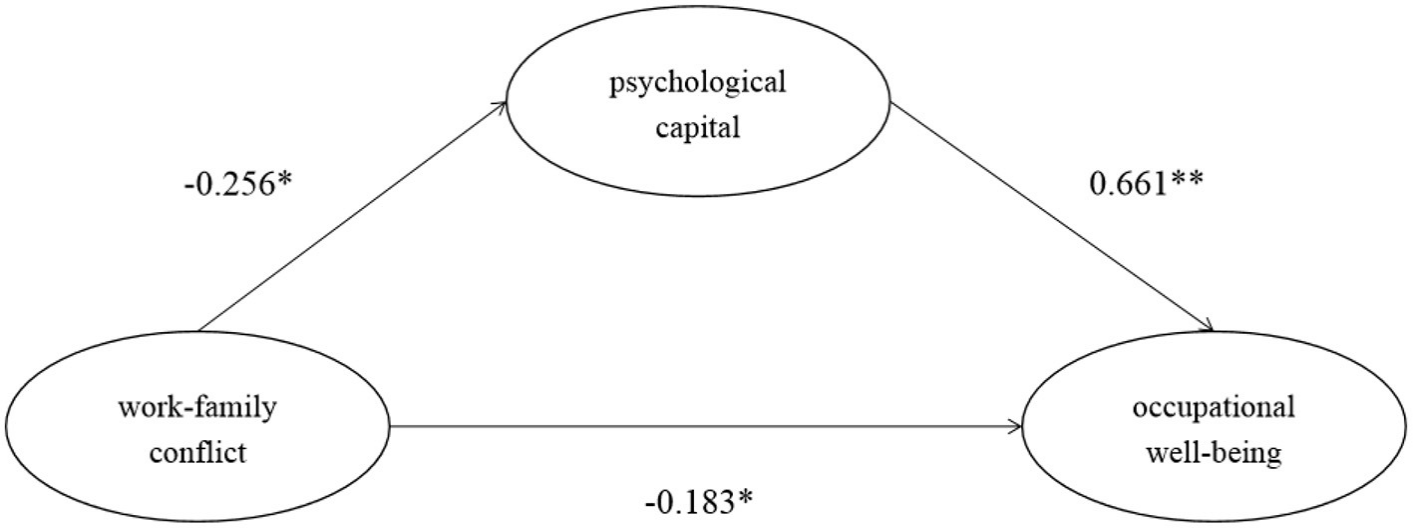
The mediating model of work family conflict, occupational well-being and psychological capital. *p < 0.05, **p < 0.01.

We used the structural equation model to test the mediating role of psychological capital in the relationship between work-family conflict and occupational well-being in Hypothesis 2. The results are shown in Figure 2 and Table 3. The total effect of work-family conflict on occupational well-being was −0.352. Moreover, the 95% confidence interval of Bootstrap with 2000 was [−0.509, −0.172], and zero was not included in this interval. Therefore, work-family conflict significantly negatively predicted occupational well-being. Hypothesis 1 was supported. The direct effect of this model was −0.183, *p* < 0.05, and the 95% confidence interval of Bootstrap with 2000 was [−0.312, −0.025]. This interval did not contain 0, so the direct effect was significant. Work-family conflict had a significant negative effect on psychological capital (β = −0.256, *p* < 0.05), and the 95% confidence interval of Bootstrap with 2000 was [−0.421, −0.044]. Psychological capital had a significant positive effect on occupational well-being (β = 0.661, *p* < 0.01), and the 95% confidence interval of Bootstrap with 2000 was [0.563, 0.757]. The indirect effect was −0.169, *p* < 0.01, and the 95% confidence interval of Bootstrap with 2000 was [−0.296, −0.036], which did not contain 0. Therefore, the mediation effect was significant, and the direct effect was also significant. Therefore, this model was a partially mediated model. So hypothesis 2 was partially supported. The fitting index of the model was (χ ^2^/*df* = 2.30, *GFI* = 0.920, *CFI* = 0.976, *TLI* = 0.954, *RMSEA* = 0.076). Each fitting index met the standard.

**Table 3:** Specific indirect effects analysis.

**Effect**	**Estimate**	**95% CI**
Indirect effect	−0.169	[−0.296, −0.036]
Total effect	−0.352	[−0.509, −0.172]
Direct effect	−0.183	[−0.312, −0.025]

**Figure 3:**
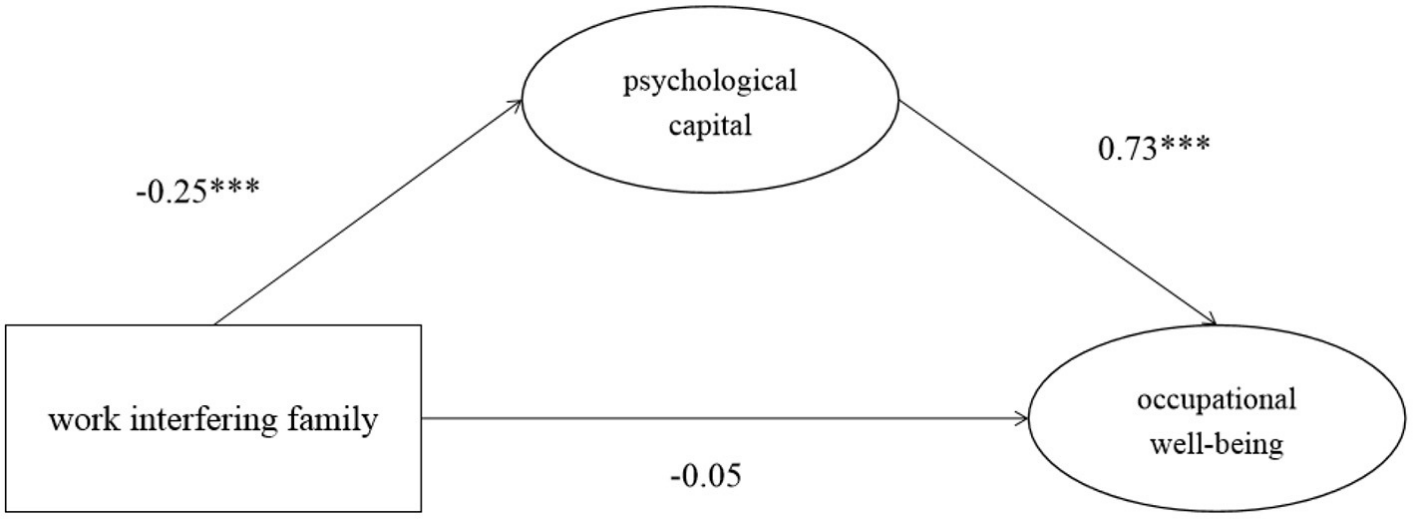
The mediating model of work interfering with family, occupational well-being and psychological capital. ***p < 0.001.

**Figure 4:**
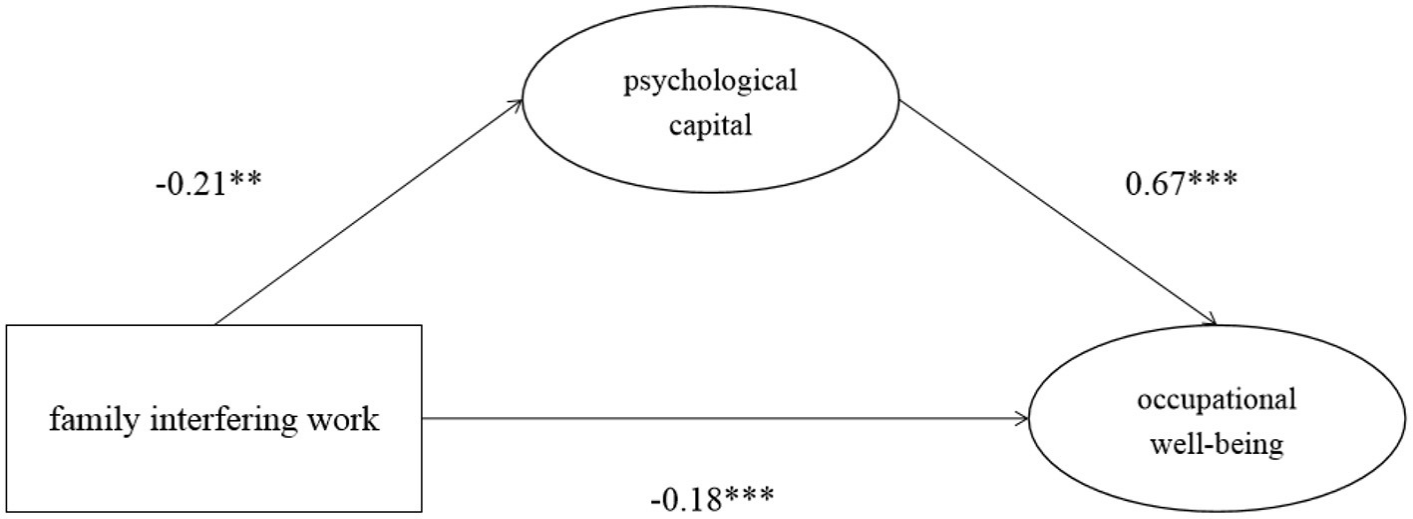
The mediating model of family interfering with work, occupational well-being and psychological capital. **p < 0.05, ***p < 0.001.

In addition, we divided the two directions of work-family conflict to test the mediating role of psychological capital in the relationship between work interfering with family/family interfering with work and occupational well-being. The results are shown in Figures 3, 4 and Tables 4, 5.

**Table 4:** Specific indirect effects analysis.

**Effect**	**Estimate**	**95% CI**
Indirect effect	−0.183	[−0.288, −0.079]
Total effect	−0.233	[−0.375, −0.070]
Direct effect	−0.05	[−0.170, 0.076]

**Table 5:** Specific indirect effects analysis.

**Effect**	**Estimate**	**95% CI**
Indirect effect	−0.141	[−0.252, −0.043]
Total effect	−0.321	[−0.448, −0.175]
Direct effect	−0.18	[−0.282, −0.035]

The total effect of work interfering with family on occupational well-being was −0.233. The 95% confidence interval of Bootstrap with 2000was [−0.375, −0.070], and 0 was not included in this interval. Therefore, work interfering with family could significantly negatively predict occupational well-being. The direct effect of this model was −0.05, *p* = 0.351, and the 95% confidence interval of Bootstrap with 2000 was [−0.170, 0.076]. This interval contains 0, so the direct effect was not significant. work interfering with family had a significant negative effect on psychological capital (β = −0.25, *p* < 0.001), and the 95% confidence interval of Bootstrap with 2000 was [−0.380, −0.105]. Psychological capital had a significant positive effect on occupational well-being (β = 0.73, *p* < 0.001), and the 95% confidence interval of Bootstrap with 2000 was [0.630, 0.816]. The indirect effect was −0.183, *p* < 0.01, and the 95% confidence interval of Bootstrap with 2000was [−0.288, −0.079]. Zero was not included in this interval, so the mediation effect was significant, and the direct effect was not significant, so this model was a full mediation model. The fitting index of the model was (χ ^2^/*df* = 2.33, *GFI* = 0.940, *CFI* = 0.920, *TLI* = 0.903, *RMSEA* = 0.077). Each fitting index met the standard.

The total effect of family interfering with work on occupational well-being was −0.321, and the 95% confidence interval of Bootstrap with 2000 was [−0.448, −0.175], which did not contain 0. Therefore, family interfering with work significantly negatively predicted occupational well-being. The direct effect of this model was −0.18, *p* < 0.001, and the 95% confidence interval of Bootstrap with 2000 was [−0.282, −0.035]. This interval did not contain 0, so the direct effect was significant. family interfering with work had a significant negative effect on psychological capital (β = −0.21, *p* < 0.01), and the 95% confidence interval of Bootstrap with 2000 was [−0.363, −0.063]. Psychological capital had a significant positive effect on occupational well-being (β = 0.67, *p* < 0.001), and the 95% confidence interval of Bootstrap with 2000 was [0.573, 0.761]. The indirect effect was −0.141, *p* < 0.01, and the 95% confidence interval of Bootstrap with 2000 was [−0.252, −0.043]. Zero was not included in this interval, so the mediation effect was significant, and the direct effect was also significant. Therefore, this model was a partially mediated model. The fitting index of the model was (χ ^2^/*df* = 1.97, *GFI* = 0.929, *CFI* = 0.948, *TLI* = 0.920, *RMSEA* = 0.066). Each fitting index met the standard.

## Discussion

### The Relationship Between Work-Family Conflict and Occupational Well-Being

In the present study, the scores of work-family conflict among primary and secondary school teachers was below the medium level, indicating that primary and secondary school teachers were troubled by work-family conflict to a certain extent. Specifically, the average score of work interfering with family was significantly higher than family interfering with work, which was consistent with the results of previous studies (3, 55, 56). Owing to the heavy workload and the large number of work hours of primary and secondary school teachers. In addition, the fact that the majority of primary and secondary school teachers were female, who had to take care of the family after work, assume the responsibilities of wife and mother, and carry out intensive teaching work, caused a degree of role conflicts.

The study also found that work-family conflict significantly negatively predicted occupational well-being. This was consistent with previous studies (12). Panatik et al. (12) found a significant negative correlation between work-family conflict and teachers' mental health, life satisfaction and subjective well-being. With the rapid development of Chinese economy, people faced with more pressure in their daily life. In addition, students in primary and secondary schools were an important structure for the country's future talents. Therefore, the task faced by primary and secondary school teachers was even more arduous. It made them impossible to balance family and work, resulting in role conflict and the decline of occupational well-being. On the other hand, when individuals devote themselves to their work (family), their energy will be consumed because of the problems and conflicts in their work (family), making it difficult for them to actively participate in family life (work). When work and family cannot be balanced, or the conflicts and problems between work and family cannot be properly handled, individuals will have anxiety, tension, and negative emotions, and their happiness will decline with the passage of time (57).

In addition, this study also examined the effects of work interfering with family and family interfering with work on occupational well-being, and explored the role of psychological capital in the relationship. The results showed that psychological capital played a full mediating role in the relationship between work interfering with family and occupational well-being, while it played a partial mediating role in the relationship between family interfering with work and occupational well-being. This result suggested that the mechanism of the effect of work interfering with family on occupational well-being was different from that of family interfering with work. According to the JDR model (35, 36), job demands refer to the physical, psychological, social, or organizational demands in work (35), work interfering with family focused on the impact of work on family, so psychological capital had a more obvious mediating effect on the relationship between work interfering with family and occupational well-being. On the contrary, family interfering with work was the influence of family on work, which had little correlation with job demands. Therefore, psychological capital had partially mediating effect on family interfering with work and occupational well-being. Researchers pointed that work and family domain factors would influence well-being. Therefore, further researches needed to focus on the mechanism of the relationship between family interfering with work and occupational well-being, for example, family domain, family satisfaction (32). In addition, it also indicated that improving the psychological capital of primary and secondary school teachers could buffer the negative effects of work interfering with family on occupational well-being. From this point of view, schools can increase teachers' life well-being, work well-being, psychological well-being, and work performance.

### The Mediating Role of Psychological Capital

This study further explored that psychological capital played a partial mediating role between work-family conflict and occupational well-being. That is, the work-family conflict of primary and secondary school teachers indirectly affected their occupational well-being through psychological capital. According to the view of positive psychology and positive organizational behavior, psychological capital was a positive psychological resource that enabled individuals to cope with stress in work and life (42, 58). In addition, based on Job Demands-Resources model (35, 38, 59), work-family conflict was one of the kind of job demands, which consumed the job resources of primary and secondary school teachers. Psychological capital, a positive psychological resource (42, 43, 60), could reduced the impact of high work-family conflict on teachers' occupational well-being. Relevant research also showed that psychological capital was an important factor to promote individual growth and development and improve job performance (61), which helped individuals to reduce the pressure and conflicts brought by work and family (62). And over time, psychological capital could improve employees' performance (41), job satisfaction (63) and employees' well-being (64). From the above analysis, increasing psychological capital was the way to decrease work-family conflict and increase occupational well-being.

### Theoretical Implication

Firstly, previous researches on well-being of employees or workers in other sectors mainly focused on single well-being, like job well-being or subjective well-being, however, the well-being should include not only job well-being or subjective well-being but also the other aspects, for example, life well-being and psychological well-being. The definition of occupational well-being included three sub dimensions: life well-being, job well-being, and psychological well-being. The present study explored the factors affecting primary and secondary school teachers' occupational well-being as a whole.

Secondly, the present study enriched the previous studies on the well-being of primary and secondary school teachers. Previous studies paid little attention to the relationship among work-family conflict, psychological capital and occupational well-being. However, this study conducted an in-depth discussion on the relationship among these three factors, and found that psychological capital played a partially mediating role in the relationship between work-family conflict and occupational well-being of primary and secondary school teachers.

Finally, it enriched the related research of the JDR model. This study took the JDR model as the theoretical basis, and the conclusions not only enriched the research on work-family conflict, psychological capital and other fields, but also enriched the JDR model. JD-R model focuses on employees' work input and broader physical and mental health indicators, such as cardiovascular disease, well-being, job satisfaction and so on (65). Moreover, JD-R model mainly explores the job demands and resources related to work stress. The work family conflict in this study belongs to work requirements, while psychological capital belongs to psychological resources in work resources (35, 36, 66), and the occupational well-being of this study is the key factor concerned by JD-R model (65). Therefore, the results of this study enrich the relevant contents of JD-R model.

### Practical Implication

In terms of practical implication, well-being was a focus that people in the whole society paid attention to, and it was also an important way for the organization to improve the job efficiency and job engagement of employees and reduce turnover intention. The results showed that work-family conflict had a negative effect on primary and secondary school teachers' occupational well-being. Therefore, schools could improve teachers' occupational well-being through reducing work-family conflict of primary and secondary school teachers. From that point of view, schools could provide the school Open Day, letting the family visit to the school, listen to the teachers' lectures and understand the teacher's work routing. In addition, the school should implement the employee assistance plan (EAP), set up courses related to work family balance, and regularly carry out marriage counseling, family counseling and parent-child counseling, so as to make the relationship between teachers and their families more harmonious and balance work and family better, so as to improve teachers' occupational well-being.

In addition, the results of this study showed that psychological capital played a mediating role in the relationship between work-family conflict and occupational well-being, that is, the higher the psychological capital was, the higher the occupational well-being would be. This provided schools a method that increasing psychological capital to improve teachers' occupational well-being, and further improve their job involvement and job satisfaction. Schools should provide teachers with EAP, set up psychological capital improvement courses, regularly carry out psychological counseling and team building activities, so as to enhance teachers' psychological capital. When teachers are full of self-confidence, hopeful for the future and able to cope with work pressure, they will experience more occupational well-being. Both direct and indirect approaches could improve teachers' occupational well-being.

The present findings should be considered in the light of the following limitations. First, this study used cross-sectional data, which only studied the relationship among the three factors, but did not deeply study the changing trend among them. Future researches can adopt longitudinal data, and explore the trend of the impact of psychological capital on the relationship between work-family conflict and occupational well-being. Secondly, as for the mechanism of work-family conflict affecting occupational well-being, only psychological capital was involved in the study, and there might still be other factors, which should be expanded in future studies. Thirdly, the sample was relatively small. We will increase the number of participants for supplementary analysis in the future.

## Conclusion

We explored the relationship between work family conflict and occupational well-being, and further investigated the mediating role of psychological capital. The results of the present study showed that psychological capital played a partial mediating role in the relationship between work-family conflict and occupational well-being, a full mediating role in the relationship between work interfering with family and occupational well-being, and a partial mediating role in the relationship between family interfering with work and occupational well-being. The present study provided a direct path (decreasing the level of work family conflict) and an indirect path (increasing the degree of psychological capital) to improve the occupational well-being of primary and secondary school teachers.

## Author's Note

Well-being was an important goal of human beings. For professional people, occupational well-being was the goal of individual pursuit. Occupational well-being included psychological well-being, job well-being and life well-being. Greenhaus and Beutell made a clear definition of work-family conflict. They pointed that work-family conflict was a kind of role conflict, in which the role of individuals played in the work (family) made it impossible to play effectively in the family (work). With the introduce of positive psychology and positive organizational behavior, psychological capital, as a positive psychological factor, might be the mechanism on which work-family conflicts affected the occupational well-being of primary and secondary school teachers. Based on JDR model, work-family conflicts as a kind of job demands, while psychological capital as a job resources, excessive work-family conflicts might consume the psychological capital energy of primary and middle school teachers and led to the decline of psychological capital. The decline of psychological capital level further led to the decline of life well-being, job well-being and psychological well-being among primary and secondary school teachers. We conducted a model to examine the role of psychological capital in the relationship between work-family conflict and occupational well-being.

## Data Availability Statement

The original contributions presented in the study are included in the article/supplementary material, further inquiries can be directed to the corresponding authors.

## Ethics Statement

The studies involving human participants were reviewed and approved by Academic Board of Shandong Normal University. The patients/participants provided their written informed consent to participate in this study.

## Author Contributions

All authors listed have made a substantial, direct and intellectual contribution to the work, and approved it for publication.

## Funding

This work was supported by National Social Science Foundation of China (Grant No. BBA200036) and Project of School of Kongzi in Shandong Normal University of China (Grant No. SDNU2019KY005). We also received for open access publication fees.

## Conflict of Interest

The authors declare that the research was conducted in the absence of any commercial or financial relationships that could be construed as a potential conflict of interest.

## Publisher's Note

All claims expressed in this article are solely those of the authors and do not necessarily represent those of their affiliated organizations, or those of the publisher, the editors and the reviewers. Any product that may be evaluated in this article, or claim that may be made by its manufacturer, is not guaranteed or endorsed by the publisher.
